# Rediscovering Pertussis

**DOI:** 10.3389/fped.2016.00052

**Published:** 2016-06-08

**Authors:** Manuela Zlamy

**Affiliations:** ^1^Department of Pediatrics, Medical University of Innsbruck, Innsbruck, Austria

**Keywords:** pertussis, whooping cough, vaccine-preventable disease, vaccination, herd immunity

## Abstract

Pertussis, caused by *Bordetella* (B.) *pertussis*, a Gram-negative bacterium, is a highly contagious airway infection. Especially in infants, pertussis remains a major health concern. Acute infection with *B. pertussis* can cause severe illness characterized by severe respiratory failure, pulmonary hypertension, leucocytosis, and death. Over the past years, rising incidence rates of intensive care treatment in young infants were described. Due to several virulence factors (pertussis toxin, tracheal cytotoxin, adenylate cyclase toxin, filamentous hemagglutinin, and lipooligosaccharide) that promote bacterial adhesion and invasion, *B. pertussis* creates a unique niche for colonization within the human respiratory tract. The resulting long-term infection is mainly caused by the ability of *B. pertussis* to interfere with the host’s innate and adaptive immune system. Although pertussis is a vaccine-preventable disease, it has persisted in vaccinated populations. Epidemiological data reported a worldwide increase in pertussis incidence among children during the past years. Either acellular pertussis (aP) vaccines or whole-cell vaccines are worldwide used. Recent studies did not detect any differences according to pertussis incidence when comparing the different vaccines used. Most of the currently used aP vaccines protect against acute infections for a period of 6–8 years. The resurgence of pertussis may be due to the lack of herd immunity caused by missing booster immunizations among adolescents and adults, low vaccine coverages in some geographic areas, and genetic changes of different *B. pertussis* strains. Due to the rising incidence of pertussis, probable solution strategies are discussed. Cocooning strategies (vaccination of close contact persons) and immunizations during pregnancy appear to be an approach to reduce neonatal contagiousness. During the past years, studies focused on the pathway of the immune modulation done by *B. pertussis* to provide a basis for the identification of new therapeutic targets to enhance the host’s immune response and to probably modulate certain virulence factors.

“I have a faint cold fear thrills through my veins” (1)

## *Bordetella* spp.

*Bordetella (B.) pertussis* is a fimbriated Gram-negative, aerobic coccobacillus. *B. pertussis* ranks to the genus *Bordetella* (2–5). Phylogenetic analysis revealed nine different *Bordetella* species. Five of them are known to cause respiratory tract infections in humans: *B. pertussis, Bordetella parapertussis, Bordetella bronchiseptica*, *Bordetella holmesii*, and *Bordetella petrii* (2, 3, 6). Within the species *Bordetella*, *B. pertussis, B. bronchiseptica*, and *B. parapertussis* are closely related pathogens that infect mammalians. *B. bronchiseptica* causes a mild or chronic respiratory infection in a large range of mammalian hosts (2, 7). In humans, it causes respiratory tract infections mostly in immunocompromised hosts (7, 8). Regarding *B. parapertussis*, two distinct hosts have been identified: humans (*B. parapertussis* HU) and sheep (*B. parapertussis* SH) (2, 9). *B. holmesii* is part of a different genetic lineage within the *B*. genus. *B. holmesii* causes either pertussis-like symptoms or invasive infections (e.g., septicemia, pneumonia, meningitis, arthritis, etc.) (10, 11). *B. petrii* was isolated in patients with cystic fibrosis and in come cases of long-lasting respiratory tract infections (Table 1) (12).

**Table 1:** **Demographic data *Bordetella* species**.

	**Phylogenetics**	**Facts**	**Infections in**
	Genus: *Bordetella*	Gram-negative 0.2–0.7 μm rods	Humans Sheep
		Phylum *Proteobacteria*	Birds
		Highly contagious	Dogs
		Obligate aerobes	Pigs
	**Human pathogen**	**Symptoms**	
Classical *Bordetella*
	*B. pertussis*	Whooping cough	
	*B. parapertussis*	Whooping cough	
	*B. bronchiseptica*	Respiratory infection	
	*B. holmesii*	Pertussis-like symtpoms or invasive infections (septicemia, pneumonia, meningitis, arthritis)	
	*B. petrii*	Respiratory tract infections	

During the past years, *B. pertussis*, the causative agent of whooping cough, resurged as cause for upper airway infections in humans.

## *B. pertussis* – Clinical Course

Infection by *B. pertussis* is acquired via droplet route (5, 13). In the susceptible child, the classical pre-vaccination textbook symptom trias is defined as: catarrhal stage with unspecific symptoms (e.g., fever, rhinitis, mild cough) which typically lasts for 1–2 weeks, followed by the paroxysmal stage where the cough evolves in the typical paroxysmal coughing spells followed by posttussive whooping and vomiting and duration of cough lasting 1–3 months. During the third stage, also known as convalescent stage, the intensity of coughing spells deceases during 1–2 weeks (14). Pertussis is at least unpleasant for the patient, as these symptoms frequently interfere with daily activities and can cause significant sleep disturbances (5, 14).

In reality, *B. pertussis* is a chameleon. Infection by *B. pertussis* nowadays often causes unspecific mild symptoms, such as rhinitis and unspecific mild cough often not leading to a physician visit (5). Even asymptomatic infections can occur in children and adults with strong residual immunity (13, 15). Life-threatening disease manifestation is often seen in newborns and young infants. Newborns and young infants often first present with apnea or respiratory distress syndromes (5, 13, 16). In <20%, fever is detected (5). The first presentation of an acute infection is affected by several parameters: patient age, previous exposure (vaccination or prior infection), first-line antibiotic administration, concomitant infections with other agents, and the presence of cross-reacting antibodies (13, 16–19).

After introduction of routine vaccination in young infants, pertussis incidence first decreased. However, *B. pertussis* nowadays accounts for a significant morbidity and mortality worldwide. Increasing incidence resulted from an increased awareness of the reservoir of *B. pertussis* infections in adolescents and adults (20–22).

Type and frequency of complications depend on host-specific age and immunity. They most commonly present as bronchoalveolar pneumonia (any age) or apnea (newborns and young infants) and more rarely as respiratory distress syndrome, seizures, and other signs of encephalopathy (2, 5).

## *B. pertussis* – Underestimated Cases?

Since the introduction of a worldwide available vaccination in the 1950s, a significant reduction in mortality rates was detected worldwide (2, 20–22). However, pertussis still poses a significant health burden. The worldwide estimated immunization coverage among infants receiving three doses of the diphtheria, tetanus, and pertussis vaccine (DTP3) increased still till 2012 and reaches about 86% of the population in 2014 (23, 24). Data on booster vaccinations are missing. Thus, the number of worldwide recognized cases of pertussis was stable, many regions reporteted a resurgence (13, 21, 23, 24). In countries with high vaccination coverage, pertussis experiences a second springtide among adolescents and adults (13, 22, 24–29). Several studies in adults revealed prolonged cough illness as a result of an infection by *B. pertussis* (13, 24–29). The United States (US) and the United Kingdom have seen a rise in *B. pertussis* cases during the past years (23, 29). The rising incidence in *B. pertussis* cases may be influenced by an either too low vaccination coverage especially booster vaccination coverage, or the possibility of a vaccination-breaktrough infection (29–32). During the past decades, improved surveillance and diagnostics has led to an increased incidence worldwide. However, in the US, a steady rise of reported pertussis cases was detected over the last 30 years (29, 30). In 2010 and 2012, pertussis outbreaks were reported in California and Washington with case counts similar to the 1940s (30). An increase across all ages also in infants less than 1 year of age has been reported in the US (33).

Recent studies tried to elucidate possible explanations for the increase of disease burden (29–32, 34–36): (1) the evolution of *B. pertussis* to escape vaccine antigens; (2) low vaccination or wild-type infection rates; (3) a changed efficacy of vaccine protection due to the use of the acellular vaccine or even a lower vaccine efficacy; and (4) an increase of reporting systems and surveillance analysis.

## *B. pertussis* – Diagnostics

For accurate diagnosis of infection due to *B. pertussis*, different diagnostic procedures are available: direct fluorescent-antibody assay (DFA), culture, PCR, and serodiagnostic.

Direct fluorescent-antibody assay is performed using nasopharyngeal swabs of patients. Via microscopy fluorescent antibodies directed against *B. pertussis* are visualized. Due to the low sensitivity and specificity of this assay, DFA diagnosis always needs a second method for proof (22, 37) Culture is the gold standard for pertussis diagnosis. Despite its low sensitivity compared to PCR, it is still used (22, 37). Nasopharyngeal samples obtained by deep aspiration or swabs can be used (22, 37–39). Collection of oral fluids is less stressful for the patient, but it should not be used for culturing due to the probable contamination with resident oral pathogens (22). In ace of culturing *B. pertussis*, addition of cephalexin to the medium is recommended to inhibit growth of contaminant bacteria (22). Agar plates are incubated at 35–37°C in a high-humidity environment with low levels of carbon dioxide for up to 12 days to reach optimal sensitivity (22). After growth on the agar plate, *bordetellae* can be further characterized by biochemical reactions, agglutination with specific sera or PCR (22, 37). During the past years, PCR assays have become a well-established method for the detection of *bordetellae* (22, 37, 40–42). Dry swabs can be used for PCR (22, 42, 43).

Serodiagnosis is often used to confirm the clinical diagnosis of pertussis. Early serodiagnostic methods required a significant (greater than fourfold) increase of titers in serum samples 2–4 weeks after the first diagnosis (22). Nowadays, enzyme-linked immunosorbent assays (ELISA) are used to differentiate IgM, IgA, and IgG antibodies against pertussis. ELISAs use specific cut-off values for detection of pertussis (22, 37, 43).

Taken together, the optimal diagnostic method always depends on the age of the patient, the stage of disease, and the primary vaccination status of the patient.

## *B. pertussis* – Virulence Factors

**Figure 1:**
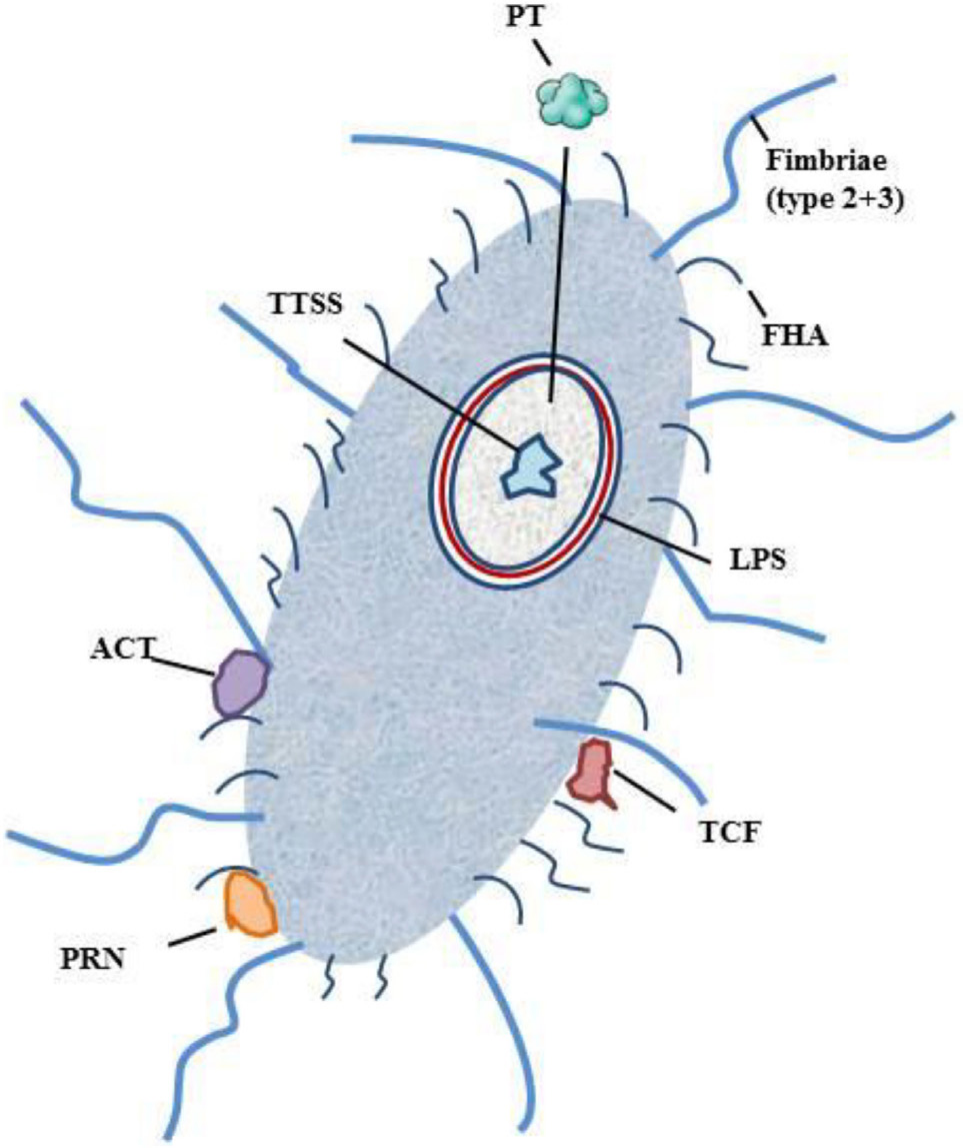
**Schematic figure of *B. pertussis* and its virulence factors**. Notation: PT, pertussis toxin; TCT, trachel cytotoxin; ACT, adenylate cyclase toxin; TTSS, type III secretion system; LPS, lipopolysaccharide; FHA, filamentous hemagglutinin; PRN, pertactin.

The primary side of infection with *B. pertussis* is the respiratory tract. Infection is initiated via contact of respiratory droplets from an infected individual (2, 3, 5, 13, 20–22). After inhalation, *B. pertussis* enters the upper respiratory tract and adheres to the epithelia of the nasopharynx and the trachea (2, 3, 5, 13, 14). After attachment, *B. pertussis* produces a cascade of virulence factors: adhesins, immune-modulators, and toxins. The interaction and teamwork of these factors prevents *B. pertussis* from a rapid clearance and enable its dissemination to the lower areas of the respiratory tract (2, 3, 5, 13, 14). *B. pertussis* produces a number of toxins: pertussis toxin (PT), tracheal cytotoxin (TCT), adenylate cyclase toxin (ACT), heat-labile toxin, type III secretion system (TTSS), and endotoxin or lipopolysaccharide (LPS). Further on receptor-binding, virulence factors, such as filamentous hemagglutinin (FHA) and pertactin (PRN), are expressed. To complete the wall of protection *B. pertussis* is protected by fimbriae, which act as antigenic targets for antibodies and T cells (Figure 1) (3, 5, 14).

Pertussis toxin is one of the dangerous players of *B. pertussis*. It promotes system effects, such as lymphocytosis and histamine sensitization, and promotes T-cell response by bystander antigens. After primary adherence by fimbriae, PT facilitates FHA-mediated adhesion to macrophages (44–46). FHA has been shown to have an immunosuppressive function during infection (3, 47). PT consists of different subunits that contribute to the immunomodulatory effects, which either suppress or promote the hosts immune response (3, 46, 48, 49). PT inhibits phagocytosis by antigen-presenting cells (APC), antigen processing and presentation, and trafficking of APC to lymph nodes (3, 46, 48, 49). TCT acts as an activator of the immune deficiency pathway (3). ACT plays several roles in the invasion of the human body by *B. pertussis*. It binds to the complement receptor 3 and intoxicates complement receptor 3-negative cells. ATC induces apoptosis and cell cycle arrest and inhibits phagocytosis, chemotaxis, and superoxide generation. Furthermore, it modulates APCs and induces a T-cell response (50–52). ACT suppresses the secretion of proinflammatory cytokines (IL-12p70) and tumor necrosis factor alpha (TNF-alpha) (53–55). FHA is the main agent for the adhesion of *B. pertussis* to the mucosal surface of respiratory tract. It promotes bacterial adherence to ciliated respiratory epithelial cells and promotes phagocytosis by macrophages and polymorphonuclear leukocytes (3, 56–60). TTSS stimulates innate and adaptive immune response (3). LPS is one of the main components for colonization survival. LPS acts pyrogenic, toxic, and can activate proinflammatory cytokine production (61, 62). PRN is an auto-transporter protein of the outer membrane that enables the adherence of *B. pertussis* to monocytes and epithelial cells (3, 63). LPS and TCT have been shown to induce NOS and NO and to inhibit DNA synthesis in epithelial cells (64) TCT and PT have been shown to inhibit the immune cell trafficking within the respiratory tract (2).

## *B. pertussis* – Immune Modulation?

After early studies on *B. pertussis*-induced immune reactions in humans, studies in mouse completed the experimental settings (65–69).

The ciliated epithelium of the respiratory tract ensures that pathogens are cleared mechanically (2). Successful infection of the host, therefore, depends on the ability of *B. pertussis* to produce a number of adhesins and toxins, which alter immune response of the host (3). After binding to the cilia of the respiratory tract, macrophages and immature dendritic cells (DCs) are the first cells responding to the invador (3, 70, 71) In addition, many toxins and virulence factors of *B. pertussis* promote bacterial survival in the host by remodulating the immune system. FHA induces proinflammatory interleukin(IL)-6 and IL-10 and supresses IL-12 production (2, 72). The generation of IL-10-producing regulatory T cells (Treg cells) suppresses interferon (IFN)-gamma production and inhibits the generation and function of Th1 effector cells (2, 72). PT promotes immunosuppression *via* activation of T-cell receptor-associated signaling molecules in lymphocytes (3). A recent study even implicates that PT can either work pro- or anti-inflammatory depending on single versus repetitive exposure of the host, which might be linked to enhanced severity of autoimmune diseases (73). In synergy with IL-10, ACT leads to the development of Treg cells, which delays the clearance of *B. pertussis* (53–55). Taken together all this cellular and humoral alterations, *B. pertussis* acts as a very potent immune modulator.

Murine infection models showed rapid cell recruitment to the lungs (74). After the initial influx of DCs and macrophages, neutrophils, natural killer (NK) cells, and T cells follow the proinflammatory signals (75, 76).

In infants with confirmed pneumonia due to *B. pertussis* infection, *bordetellae* have been found in pulmonary alveolar macrophages (71). *B. pertussis* can replicate in macrophages and, therefore, evade destruction (3, 70). As a consequence, depletion of resident macrophages enhances infection (70). Controversely, former studies revealed that macrophages can harbor *B. pertussis* intracellularly and then be activated by IFN-gamma and IL-17 to kill the intracellular *B. pertussis* particles (77, 78). The second first-line immune cells activated are DCs. DCs present antigens to T cells and stimulate innate cytokines that promote further differentiation of naive T cells. After recognition of *B. pertussis*, proinflammatory signals (Il-12, IFN-gamma) trigger activation of T-cell response (79). In human DC cells, infection by *B. pertussis* enhances IL-1 and IL-23 production, which is required for maturation of Th17 cells (80).

In mouse models, neutrophils, which help to kill phagocytosed bacteria, infiltrate the lungs in around day 5 after infection (3, 81). Due to its unique structure, *B. pertussis* can survive in neutrophils that undergo lysosomal maturation (58). During the early time of infection, PT delays the early infiltration of neutrophils (82) and ACT inhibits neutrophil functions like phagocytosis, superoxide generation, and chemotaxis (83). Another early player of the defense against *B. pertussis* is NK cells. NK cells produce IFN-gamma in response to infection and lead to a Th1-guided immune response (76, 84).

In a second defense line of the human body, proteins are secreted by the mucosa of the airways and by innate immune cells: lysozyme, lactoferrin, and secretory leukoproteinase inhibitor, and antimicrobial peptides (AMPs), e.g., cathelicidin (LL-37) and defensins (85, 86). *B. pertussis* fights against these agents by blocking certain molecules. For example, TTSS inhibits the expression of defensins and, therefore, promotes survial of *B. pertussis*. Consequentely, *B. pertussis* is enabled to colonize the lower airways (87). *B. pertussis* also has mechanisms protecting more or less against another soluble factor of the innate immune system: the complement. Susceptibility to complement remains highly variable (88).

On the cellular level, certain players are involved. Recent studies detected that cellular components of the immune system are needed to effectively clear a primary infection by *B. pertussis*. CD4− T cells, Treg, and Th17 cells seem to play a crucial role in the pathogenesis of pertussis (89, 90). Early work focusing on the T-cell immune system evaluated that PT, FHA, and PRN stimulate CD4+ T cells in children with whooping cough (65–67). *In vitro* proliferation of T cells negatively correlates with clinical symptoms of pertussis (68). In murine models, high levels of CD25−Foxp3+ Treg cells have been detected in the lungs of infected animals (91). Recent studies showed that T-cell response plays a major role in protection against *B. pertussis* (89). It is assumed that the high amount of Treg cells may be a benefit to the infected patient by limiting pathological alterations (78).

More recent studies showed an induction of Th17 cells by *B. pertussis* (78, 80). Pulmonary hypertension is one possible lethal complication of pertussis infection in infants and young children. Interestingly, Th17 cells are discussed to contribute to the pathomechanisms of pulmonary hypertension in severe pertussis cases (92). By the induction of Treg cells, pertussis subverts the protective immune response (72, 93). During infection, protective Th1 and Th17 response can be detected locally and systemically (94). However, the exact role of Th17 cells in protection against *B. pertussis* has to be more precisely studied in humans.

Despite all achievments, the first priority for the improvement of a long-lasting protection after vaccination is to study the exact immunological responses to infection and identify new targets that improve the robustness of pertussis vaccination. Furthermore, the highest mortality rates are known in infants. The infantile immune system is difficult to treat and protect. Considering these challenges, future studies should focus on new priorities irrespective of the socioeconomic status of the patients.

## *B. pertussis* – Vaccinations

Up to date, two differenct vaccines can be used: an acellular pertussis (aP) vaccine and a whole-cell pertussis (wP) vaccine. Early studies in murine models and humans have revealed that wP and aP vaccine induce distinct Th1 versus Th2 responses (44, 45, 95). In case of the aP vaccine, the T-cell immune responses to pertussis were assessed during the safety and efficacy trials conducted in Sweden and Italy in the 1990s (96, 97). In these early studies, a “robust” T-cell immune response to the pertussis vaccine was detected in infants and young children (96, 97). Following studies showed that T-cell immunity persisted over a long time period even after the decline of antibodies (98, 99). Furthermore, T-cell immunity could be boostered by wild virus infection (99). By contrast, T-cell response after wP vaccine was compareable to natural infection, inducing a Th1 response (98, 100, 101). After primary vaccination with wP vaccine, an aP booster dose induces a mixed Th1/Th2 response (44, 45).

Several studies emphazised the importance of booster vaccinations to enhance the T-cell response to pertussis antigen. In a study in adolescents, Rieber et al. pointed out that T-cell parameters to PT, FHA, PRN, and fimbriae increase after booster vaccination with a five-component Tdap booster vaccine (102). Due to lacking immunity more recently, a more complicated understanding of immunity after pertussis aP vaccination occurred. An aP booster vaccination in preterm infants between 13 and 16 months of age did not induce a significant immune response after vaccination, when compared to values before the booster vaccination (103). Similarly, another study showed that in children who were first vaccinated with aP vaccine an increase in cytokine production was missed after booster vaccination, whereas children who were first immunized with wP vaccine did show an increase in cytokine production (104). In children at 9 years of age, a second aP vaccine booster dose did not increase T-cell respose (105). One possible explanation might be that the enhancement of T-cell immunity during the 5 years following the booster at 4 years of age is probably caused by natural boosting (104). Early studies also proofed that vaccination-induced T-cell response could wane by 4 years of age and can be naturally boosted by symptomless wild-type infection (98). Another possible explanation for the differing results might be the differences in study design. While the early studies took blood samples to measure immune responses from the same subject before and after vaccination (102, 106), more recent studies had different subjects in the boostered and non-boostered study group (104, 105).

Nowadays, it is discussed if the duration of immunity of aP vaccines in the 1990s was overestimated due to an natural booster because of high wild-type pertussis infections. It is speculated that wild-type infection and subclincal pertussis infection may induce a long-term immunity in previously infected or immunized individuals (3, 89).

**Figure 2:**
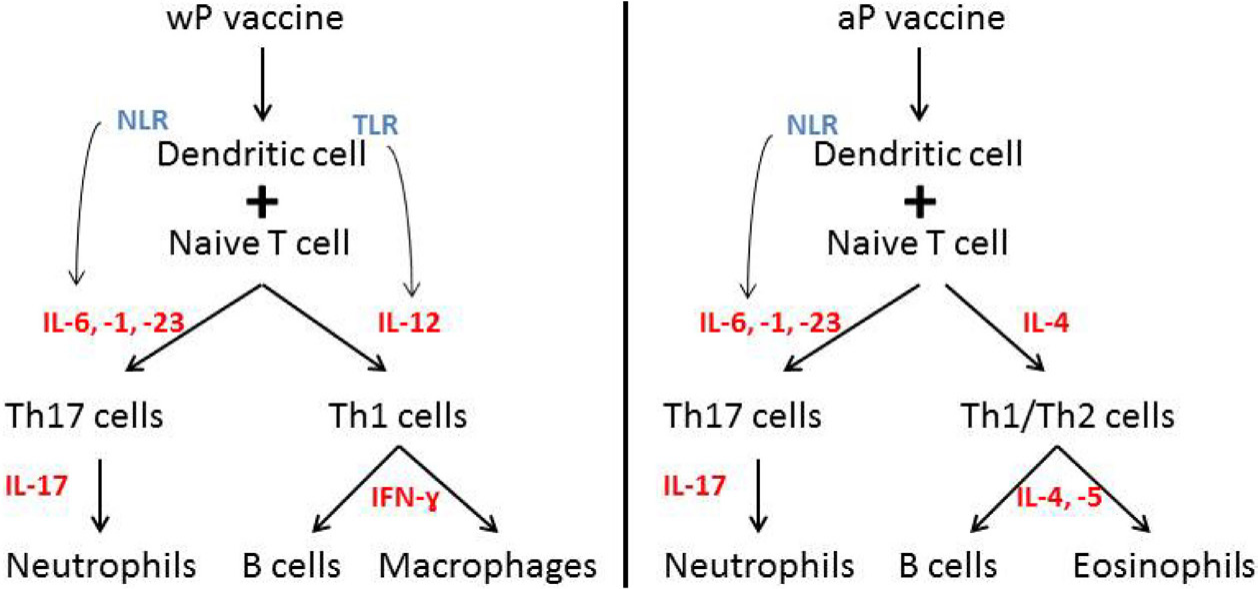
**Immue response after wP versus aP vaccination**. Different immune responses after wP versus aP vaccination. Distinct cytokine induction leads to the induction of different immune cells. Notation: IL, interleukin; NLR, NOD-like receptor; TLR, Toll-like receptor.

Immunization of children with wP induced a CD4+ and CD17+ T-cell respone (3, 107). wP vaccines include pathogen-associated molecular patterns (PAMPs) (e.g., LPS) that induce a IL-1, -6, -12, and -23 production by macrophages and DCs (3). By contrast, vaccination with aP was shown to induce a TH2 or Th1/Th2 response (3, 107). aP vaccines consist of the adjuvant alum, which stimulates IL-1, IL-4, or IL-17 (3). When wP and aP are compared, different cytokines are stimulated after vaccination promoting the induction of different T cells and B cells (3) (Figure 2). Recent data showed that after primary aP vaccination, CCR7+CD45RA− (central memory 328 T cells) and CCR7−CD45RA− (effector memory T cells) T-cell subsets are induced (108, 109). It is discussed that, after vaccination, a greater amount of central memory T cells is associated with greater amount of Th1 cytokines after infection, whereas a greater amount of effector memory T cells is more likely associated with a Th2 response (110). Former studies showed that pertussis-specific CD8+ memory T cells are induced after vaccination (111), but booster vaccination had no effect on the total number of these specific T-cell subsets (109).

According to vaccine-specific long-term protection against pertussis, the available studies are problematic to compare. Data from different geographic areas with specific pertussis epidemiology and differences in the methodology used are hard to compare. Second, the determination of an asymptomatic natural booser is hard to predict. As a consequence, many studies on different vaccines and vaccination schedules in a variety of countries exit. A study in a pre-adolescent cohort showed that wP vaccination during infancy induced a longer lasting T-cell immunity than aP vaccination (109). This study showed that *in vivo* cytokine response to antigenic stimulation was higher in subjects who received wP vaccination even if the time from the last booster dose was significantly longer than in aP vaccinated subjects (109). Controversially, other studies showed that the antigen-specific cytokine response improved after shift from wP to aP vaccination (104, 105). Interestingly, studies of the past years unveiled that protective immunity obtained after aP vaccination wanes more rapidly than after wP vaccination (112). To overcome these deficiencies, many efforts are in progress, e.g., the inclusion of additional antigens in aP vaccines, the reformulation with adjuvants that more favor Th1 and Th17 cell response, and the development of live-attenuated vaccines (113). The development of a live attenuated vaccine has several advantages, including the generation of a mucosal immunity. However, it remains unclear if a brought public acceptance will be reached. Therefore, it should be considered to retain the immunogenicity of wP vaccines. One has to keep in mind that the development and approval of a new vaccine will be a long-lasting process.

However, it is still unclear which vaccination strategy might be the most effective. Actual studies on vaccination effectiveness and population-based vaccine coverage rates are not comparable. Therefore, it is not possible to identify a predictive value for the estimated vaccination coverage. Further studies should use comparable vaccination and testing schedules in age matched patients and controls for a more precise estimation of real duration of vaccination coverage.

## *B. pertussis* and Aging?

Pertussis affects all people from the first hour of life to the last breath. Throughout life, the immune systems undergo several changes that might lead to age-related difference in the pertussis-specific immune response. During the past years, more insight was gained into vaccine-specific B- and T-cell memory. With ongoing age, significantly stronger waning of vaccine-induced memory B cells is detected when compared to younger age groups (66). Studies in infants detected a mature development of Th1 and Th2 response in neonates and pre-terms (114, 115). With ongoing age, the lymphoproliferative responsiveness is lost (66, 110). Taken together, studies showed an impact of immunosenescence on pertussis-specific immunity via a decreased T-cell responsiveness (66).

## *B. pertussis* – Cocooning Versus Vaccination During Pregnancy

After the resurgence of pertussis infection, several studies showed that the main source of infection in newborns and infants were close contact persons, mostly family members (116, 117). In a first attempt to reduce pertussis incidence, indirect protection by reduction of transmission rates was favored, as the so called “cocooning strategy.” Therefore, some countries adapted their national immunization guidelines (116, 118, 119) and some studies were elicited. Another study focused on the influence of vaccination rates among siblings and vaccination rates among mothers showed that the provided protection rates are comparable (120). A recent study on the effect of cocooning infants younger than 6 months of age did not detect any reduction in pertussis cases among infants younger than 6 months of age (117). It is discussed controversially if cocoon strategies are cost-effective or even prevent infections (116, 121, 122). Taken together, it is advisable for women to know their immunization status and to identify all close contact persons (family members, non-household close contact persons), which may play a considerable role in the transmission of pertussis.

Another attempt to reduce pertussis rates among newborns and young infants was the introduction of pertussis vaccinations during pregnancy. Vaccination during pregnancy has become more important in some countries.

Up to date pertussis cocooning strategies remain deficient and vaccines are licensed for use after 6 weeks of age (116, 123–125). Due to a steady transplacental transfer of pertussis antibodies from the mother to the fetus, health authorities first recommended in 2011 the use of pertussis vaccinations for pregnant woman (126–128). The US first recommended maternal vaccination after gestational week 20 and subsequently the time window was narrowed to gestational week 27–36 (129). Switzerland and the United Kingdom adopted these recommendations (128). Early studies showed that vaccination with Tdap vaccines during gestational week 27–30 + 6 was associated with the highest values of IgG in umbilical cord blood when compared to vaccination beyond gestational week 31 (125). According to one of the most potent virulence factors of pertussis PT (44–46), it was shown that vaccination of the mother between gestational week 27–30 + 6 elicited the highest PT antibody concentrations at birth (125). A recent study supports these data because it showed that maternal Tdap vaccination in the early second-trimester significantly increases neonatal antibodies at birth when compared to third-trimester vaccinations (123). All in all the antenatal vaccination campaign in the United Kingdom achieved a vaccine coverage of 60% with >90% effectiveness (130, 131). A recent study in the United Kingdom showed that after introduction of pertussis vaccination during pregnancy a strong reduction in confirmed cases and hospital admissions because of pertussis, especially in infants younger than 3 months of age was reported (131). Furthermore, the question arose if vaccination early in pregnancy might adversely affect the infant’s immune response to vaccinations after birth. Some studies showed that antibody concentrations at birth did not interfere with the immune response to further immunizations after birth (132–134). It is known that maternally derived antibodies are able to interfere with the infant’s immune responses with the same vaccination (135), which was detected after DTaP vaccination (136). It was shown that maternal antibodies interfere with antibody responses after primary vaccination during infancy in children born to Tdap-vaccinated mothers (127). Interestingly, a mouse model showed that the vaccination of infant mice reduced the protective functions of maternally derived antibodies *in vitro* and *in vivo* (137). A study that focused on the Repevax vaccination (a combined tetanus, low-dose diphtheria, 5-component aP, inactivated polio vaccine; Repevax; Sanofi Pasteur) detected a significant attenuation of pertussis antibodies in infants whose mothers where vaccinated with Repevax during pregnancy (136).

Spotlighted by recent findings, the lack of protection by aP vaccines, the efficacy of current vaccines should be maximized by prenatal vaccination, additional boosting, and alternative vaccination strategies. In future, it is important to determine the functionality of maternal and infant antibodies to better understand a probable interference of vaccination during pregnancy and later vaccinations of the infants.

## *B. pertussis* – Conclusion

It is irrefutable that the incidence of severe pertussis cases is rising worldwide. Nearly 90% of all cases of deaths caused by pertussis occur in infants younger than 4 months of age (113). Most of these cases are caused by fatal pertussis pneumonia caused by PT (113). Therefore, it is also imperative to conduct studies focusing on the limitation of PT activity during acute infection. During the past years, the resurgence of pertussis lead to many new studies focusing on a better understanding of transmission dynamics, virulence factors, and host immune reactions as well as the search for new vaccine targets. It was shown that the first tries to achieve herd immunity and focusing on cocooning and possible eradication failed. It is discussed if a meanwhile switch to wP vaccine as a first dose in the primary immunization schedule should be introduced (29). Frightfully by gaining more and more inside into the cellular and humoral immune response to an infection caused by *B. pertussis*, more and more questions arise. Efficacious vaccines need to be long-lasting, prevent transmission, and reduce disease burden. Up to date, none of the existing vaccines fulfils these criteria. Recent studies highlighted that a likely effective immune response requires the induction of a Th1/Th17 immune response, which stimulates opsonizing, toxin-neutralizing, and mucosal antibody production as well as the induction of a memory T-cell response, which recruites and activates phagocytes. Therefore, it is an urgent need to re-evaluate certain immunization routes to achieve a better vaccine. New studies on vaccinations during pregnancy showed interesting first results but long-term protection in the new borne have to be controlled over time. Furthermore, more detailed studies on the surveillance rates of symptomatic and asymptomatic infections and the examination of the genetic diversity of circulation *B. pertussis* strains may probably lead to a better understanding of possible prevention targets.

Although all insights into the pathogenicity of pertussis infection identified in animal models, our understanding of the human disease has to be improved. Therefore, more detailed studies on several levels, including gene expression, virulence-factor delivery, binding-specificity and activity have to be conducted. Because we should not forget, that we still do not know why infected patients cough!

## Author Contributions

The author confirms being the sole contributor of this work and approved it for publication.

## Conflict of Interest Statement

The author declares that the research was conducted in the absence of any commercial or financial relationships that could be construed as a potential conflict of interest.
